# Promising efficacy of teniposide in H3K27M mutant diffuse midline gliomas: a case report

**DOI:** 10.3389/fonc.2026.1784250

**Published:** 2026-04-16

**Authors:** Xiaoman Wang, Tingting Niu, Junxian Xia, Jing Liu, Mengqi Sun

**Affiliations:** 1 The Second Clinical Medical College of Jinan University, Department of Radiotherapy, Shenzhen People’s Hospital, Shenzhen, Guangdong, China; 2 The Second Clinical Medical College of Jinan University, Department of Pharmacy, Shenzhen People’s Hospital, Shenzhen, Guangdong, China; 3 Departments of Pathology, Shenzhen Second People’s Hospital, The First Affiliated Hospital of Shenzhen University Health Science Center, Shenzhen, Guangdong, China

**Keywords:** chemotherapy, diffuse midline glioma, H3K27M mutation, targeted therapy, teniposide

## Abstract

Diffuse midline glioma with H3K27M mutation (H3K27M-mutant DMG) is a rare central nervous system tumor with an extremely poor prognosis. The absence of a standard treatment regimen makes achieving durable survival benefits a major therapeutic challenge. This study reports the case of a 17-year-old male diagnosed with H3K27M-mutant glioma. Following surgical resection and postoperative concurrent chemoradiotherapy, the patient received adjuvant therapy combining teniposide (VM-26) and bevacizumab, which led to significant radiological and symptomatic improvement. By examining the efficacy of this individualized comprehensive strategy—integrating surgery, chemoradiotherapy, and targeted therapy with teniposide—we assess its potential mechanisms and clinical value. This case suggests a potentially effective treatment option for patients with H3K27M-mutant DMG.

## 1 Introduction

Diffuse midline glioma (DMG) is a rare yet highly aggressive central nervous system tumor, with the H3K27M-mutant subtype being a defining entity (1). H3K27M-mutant DMG predominantly affects children and adolescents, showing a peak incidence in those aged 5–10 years. DMGs account for 10-20% of all pediatric brain tumors (2). According to the 2021 WHO classification, this tumor is categorized as a grade IV, IDH-wildtype, H3K27M-mutant glioma. It carries a dismal prognosis, with a two-year survival rate below 10% and a median overall survival (OS) typically less than 12–15 months (3, 4). The tumor’s frequent location in eloquent midline structures such as the brainstem, thalamus, and spinal cord makes complete surgical resection challenging. Postoperative radiotherapy is the first-line treatment, but its efficacy as a standalone modality is limited (5). Currently, no standard systemic therapy exists, creating an urgent need to explore safe and effective systemic treatment strategies.

In this case, following surgery, the patient received concurrent chemoradiotherapy with temozolomide (TMZ) combined with bevacizumab. However, a follow-up magnetic resonance imaging (MRI) scan two months post-radiotherapy showed minimal tumor regression. Subsequently, teniposide was added to the regimen alongside temozolomide and bevacizumab. After five cycles of this modified therapy, a follow-up MRI demonstrated a marked reduction in tumor enhancement. This was accompanied by significant symptomatic improvement, including alleviation of left-sided limb weakness and numbness, with no new metastatic lesions observed. Teniposide, a drug used in malignant brain tumor therapy, offers favorable pharmacological properties such as high lipophilicity and good blood-brain barrier penetration, and retains efficacy in high-grade gliomas (6). In this patient, the combination therapy effectively reduced tumor enhancement, achieving both radiological and clinical symptom relief. The regimen demonstrated favorable efficacy and safety, with no severe or unmanageable adverse events. This case provides compelling evidence for the antitumor activity and acceptable toxicity profile of teniposide in H3K27M-mutant DMG, offering valuable insights to guide its further research and clinical application.

## 2 Case description

**Figure 1:**
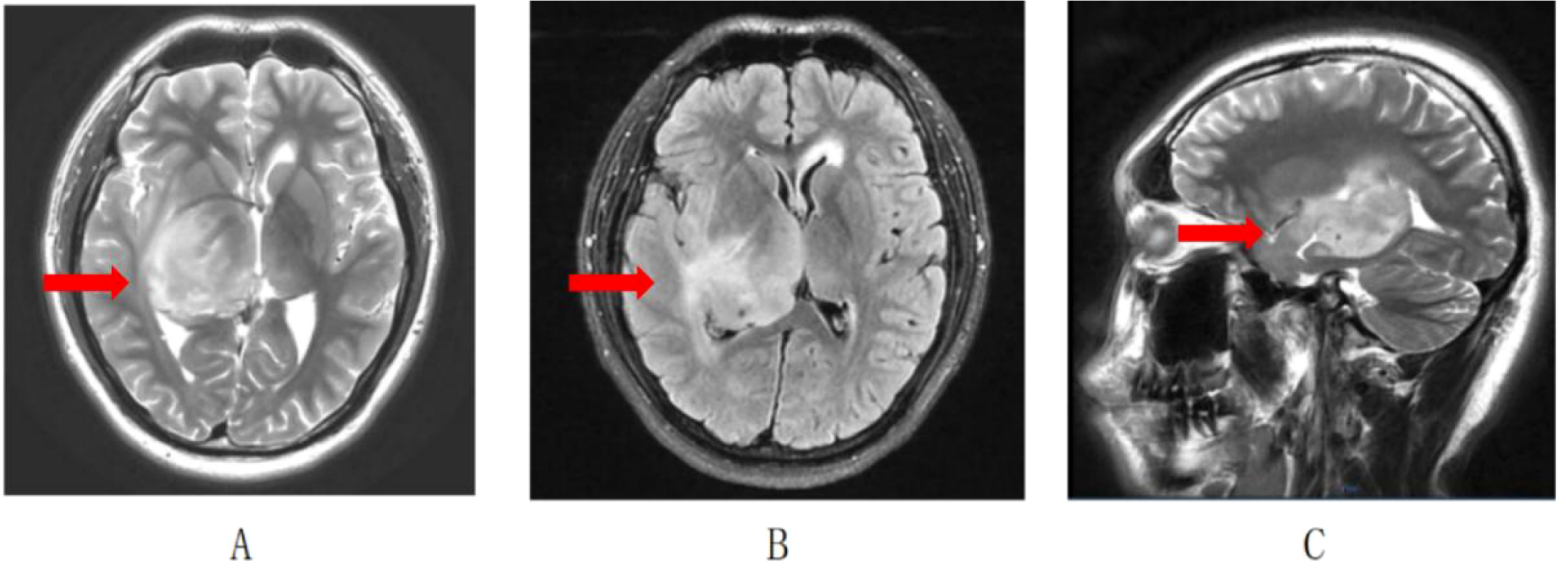
MRI revealed a large mass lesion in the right thalamus and basal ganglia. **(A)** T2 image; **(B)** T2 FLAIR image; **(C)** T2 sagittal view.

A 17-year-old male presented to the Sun Yat-sen University Cancer Center with a three-month history of left-sided facial and limb numbness. A brain magnetic resonance imaging (MRI) scan performed on March 14, 2025, revealed extensive abnormal signals involving the right temporo-insular region, thalamus, and periventricular areas around the left lateral and third ventricles, suggestive of a diffuse glioma accompanied by cerebral herniation(**Figure 1**). After the surgical team discussed the associated risks and potential complications, the patient’s family declined surgical intervention due to the perceived high risk. Supportive care, including measures to reduce cerebral edema, was initiated, leading to mild improvement in the limb numbness.

**Figure 2:**
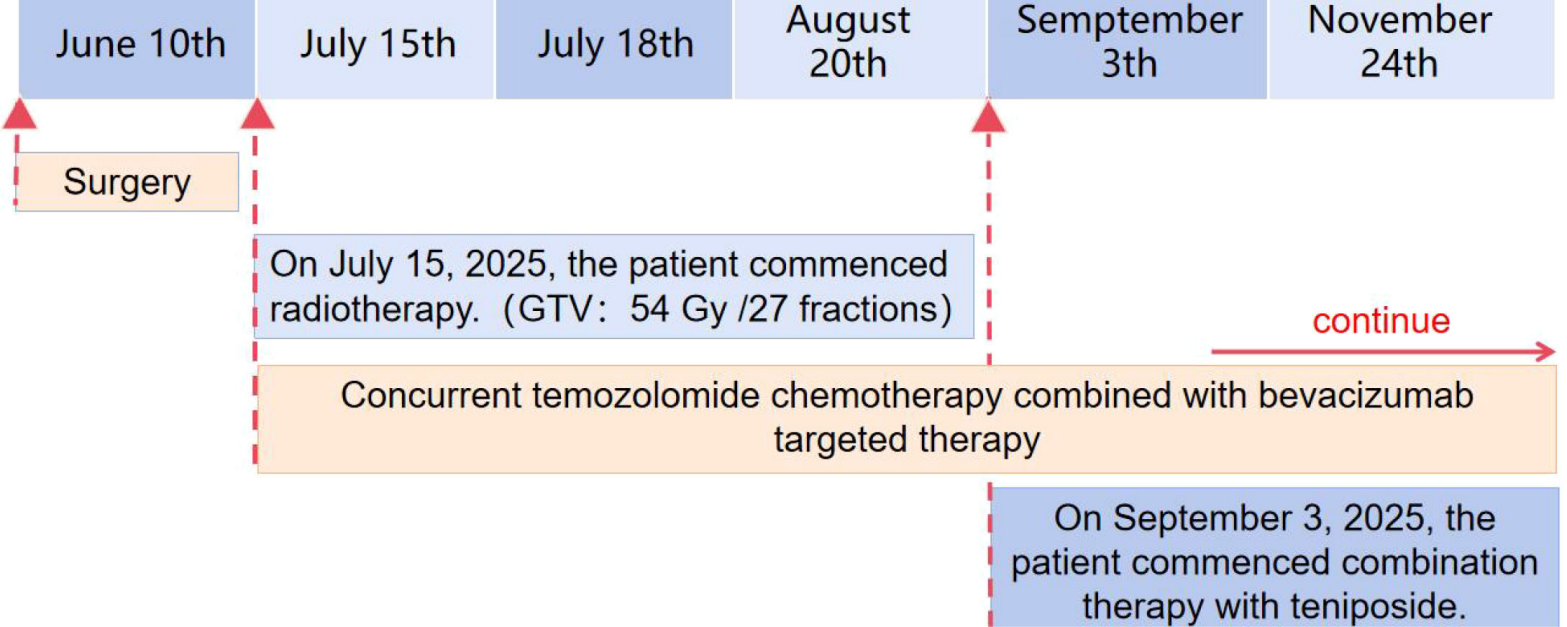
A timeline with relevant data from the episode of care.

In May 2025, the patient presented to the Shenzhen Second People’s Hospital with worsening symptoms, including subjective visual decline and increased left-sided limb weakness. Ophthalmological examination identified a right visual field defect and left temporal hemianopia. Based on the clinical presentation and imaging findings, and with informed consent from the family, the patient underwent a right thalamic-brainstem tumor resection via craniotomy on June 10, 2025 (**Figure 2**).

**Figure 3:**
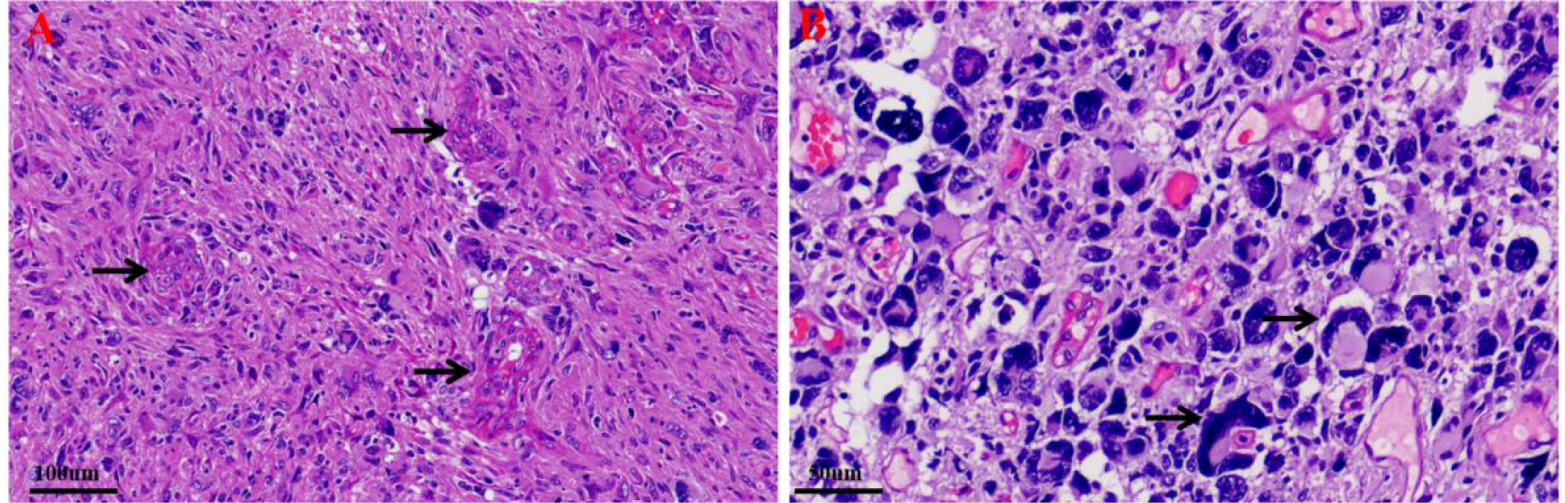
Histopathological characteristics of the tumor. Hematoxylin and eosin (H&E) staining of the resected lesion (March 2025) revealed a hypercellular glial neoplasm. The tumor was composed of spindle-shaped to giant cells exhibiting an increased nuclear-to-cytoplasmic ratio. Frequent mitotic figures and microvascular proliferation were evident. **(A)** Original magnification, ×200;Scale bar:100μm. **(B)** Original magnification, ×400;Scale bar:50μm.

**Figure 4:**
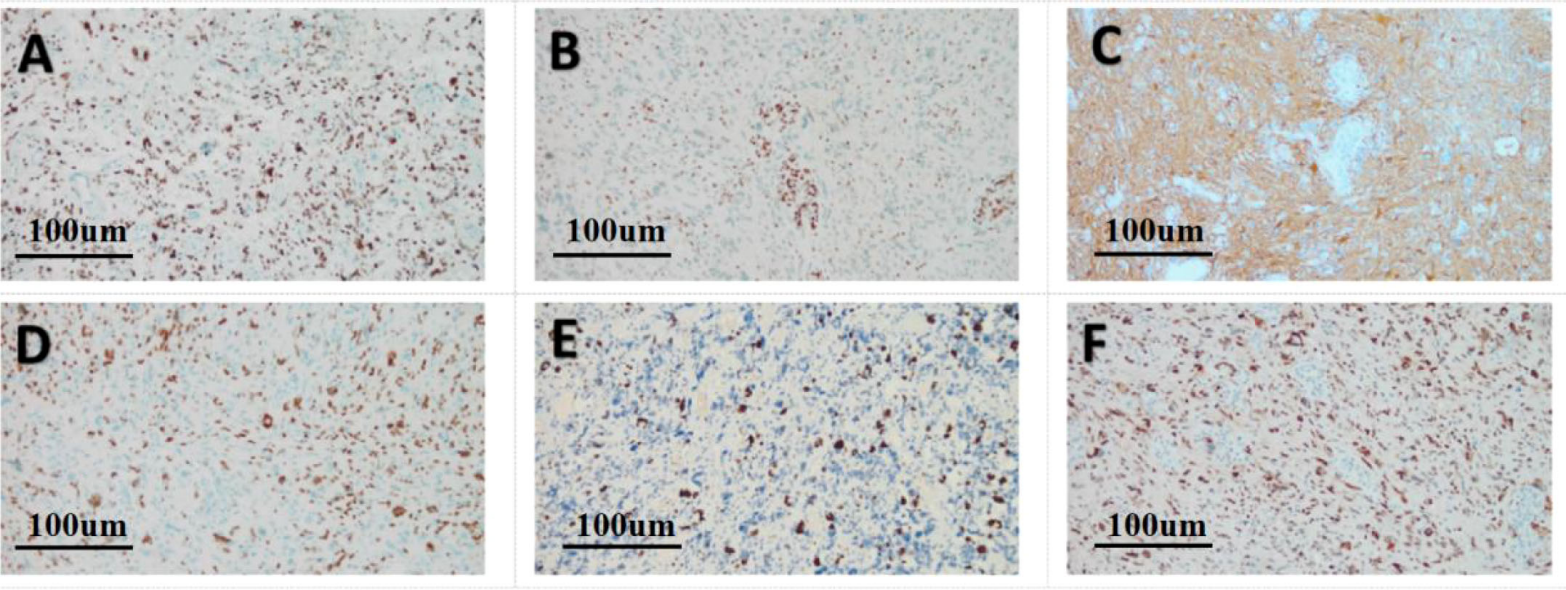
Immunohistochemistry results(original magnification, x200; Scale bar:100μm). **(A)** H3K27M staining was positive. **(B)** H3K27me3 staining was negative. **(C)** GFAP was positive. **(D)** O1ig2 was positive. **(E)** Ki-67 was 40% positive. **(F)** P53 was 90% positive.

Histopathological examination of the surgical specimen revealed a hypercellular glial neoplasm composed of spindle-shaped to giant cells exhibiting an increased nuclear-to-cytoplasmic ratio, frequent mitotic figures, and microvascular proliferation (**Figure 3**). Molecular profiling demonstrated no mutations in BRAF V600E, isocitrate dehydrogenase (IDH), and the telomerase reverse transcriptase (TERT) promoter. There was no 1p/19q co-deletion or CDKN2A homozygous deletion. A mutation in the H3F3A gene (p.K27M) was detected. Immunohistochemistry (IHC) (**Figure 4**) was positive for H3K27M and glial fibrillary acidic protein (GFAP), with scattered cells positive for Oligodendrocyte lineage transcription factor 2 (Olig2). Staining was negative for H3K27me3, IDH1 R132H, BRAF V600E, and CD34. The tumor retained expression of Alpha-thalassemia/mental retardation syndrome X-linked (ATRX) protein and DNA mismatch repair proteins (MLH1, MSH2, MSH6, PMS2). Ki-67 was 40% positive, and P53 was 90% positive. (**Figure 4**). Molecular profiling performed at an external institution revealed an unmethylated status of the O^6^-methylguanine-DNA methyltransferase (MGMT) gene promoter. The integrated histopathological diagnosis was diffuse midline glioma, H3 K27-altered, World Health Organization (WHO) grade 4.

Given the large tumor size and subtotal resection, the patient proceeded to adjuvant chemoradiotherapy combined with targeted therapy. Intensity-modulated radiation therapy (IMRT) was initiated on July 15, 2025. In accordance with high-grade glioma contouring guidelines, a dose of 54 Gy in 27 fractions was delivered to the gross tumor volume (GTV), while the clinical target volume (CTV) received 50.22 Gy in 27 fractions. Concurrent oral temozolomide chemotherapy was administered at a dose of 140 mg (75 mg/m²) daily under fasting conditions. Targeted therapy with bevacizumab (400 mg per cycle) was initiated on July 18, 2025; a total of 10 cycles were administered, completed by November 27, 2025.

**Figure 5:**
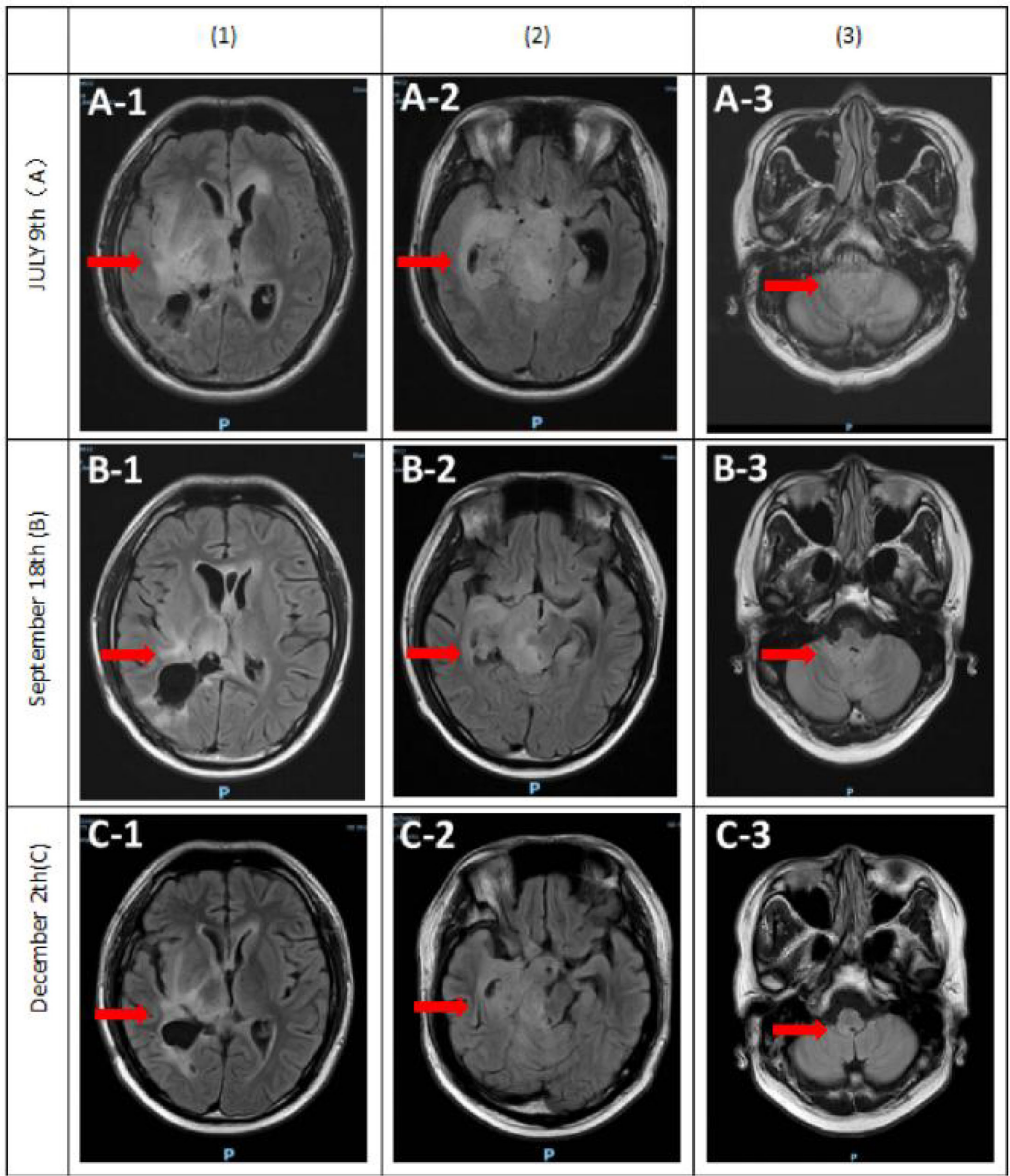
Brain MRI (T2 FLAIR Images). (A1-A3)Postoperative MRI following subtotal tumor resection. (B1-B3) Follow-up MRI obtained over two months (September 18) after concurrent chemoradiotherapy and targeted therapy, showing mild reduction in lesion size. (C1-C3) Follow-up MRI (December 2) after five cycles of combined teniposide, temozolomide, and bevacizumab therapy, demonstrating marked tumor regression and decreased midline shift.

A follow-up MRI on September 18, after completing the concurrent chemoradiation, showed only marginal tumor reduction. The patient’s neurological symptoms, including limb numbness and weakness, showed no significant improvement. Considering the tumor’s unmethylated O^6^-methylguanine-DNA methyltransferase (MGMT) promoter status, which is associated with limited efficacy of temozolomide, the adjuvant regimen was modified. Beginning September 3, 2025, the patient received combination therapy consisting of teniposide (100 mg), temozolomide, and bevacizumab. He completed five cycles of this regimen by November 24, 2025. A subsequent MRI on December 2 demonstrated significant tumor regression (**Figure 5**), accompanied by marked improvement in his limb numbness and weakness. The patient remains on continued adjuvant therapy immunotherapy.

## 3 Discussion

In 2016, the World Health Organization (WHO) introduced a new diagnostic entity in its classification of central nervous system tumors: diffuse midline glioma, H3 K27M-mutant (7). This revision underscored the critical role of molecular markers in defining prognosis and guiding treatment. The subsequent 2021 WHO classification further categorized pediatric diffuse gliomas primarily into high-grade and low-grade types. Major high-grade entities include diffuse midline glioma (H3 K27-altered), diffuse hemispheric glioma (H3G34-mutant), diffuse pediatric-type high-grade glioma (H3-wildtype and IDH-wildtype), and infant-type hemispheric glioma (8). Diffuse midline glioma with H3K27M mutation predominantly affects children and adolescents, 80% of pediatric thalamic GBM tumors harbor the H3K27M substitution (9). These tumors typically arise in these structures such as the brainstem, thalamus, and spinal cord (10).

Their defining molecular feature is a lysine-to-methionine substitution at position 27 of histone H3 (H3K27M). This mutation leads to a global reduction in H3K27 trimethylation, a concomitant relative increase in H3K27 acetylation, and widespread dysregulation of oncogenic gene expression, which drives the tumor’s aggressive behavior (11). The presence of the H3K27M mutation is strongly associated with a very poor survival rate, although the precise prognosis depends on various factors (12). Patients with H3K27M-mutant DMG typically have a median overall survival of less than one year and a two-year survival rate below 10% (13, 14). The prognosis appears worse for patients with H3.3 K27M mutations compared to those with H3.1 or H3.2 K27M mutations or with EZHIP overexpression. Investigated treatment modalities include surgical resection, radiotherapy, chemotherapy, targeted therapy, tumor-treating fields, and immunotherapy (15).

The highly aggressive nature of H3K27M-mutant DMG presents a major therapeutic challenge. The primary surgical goal is maximal safe resection. However, when tumors involve critical midline structures like the brainstem or thalamus, the associated surgical risks are substantial, often limiting the procedure to a subtotal resection for cytoreduction (16). Postoperative radiotherapy remains the standard of care, particularly for patients, like the one presented here, in whom complete resection is not feasible (17, 18). Radiotherapy utilizes high-energy X-rays or other forms of radiation to eliminate tumor cells within the primary site and a margin of surrounding tissue. It is an established treatment modality that provides definitive clinical and radiographic benefit. Nevertheless, optimal radiation target volumes and dose prescriptions are not yet firmly established. The 2022 Chinese Anti-Cancer Association Guideline for the Integrated Diagnosis and Treatment of Glioma recommends focal radiotherapy at doses of 54–60 Gy, as well as the Stupp protocol of concurrent chemoradiotherapy (19). The 2025 Expert Consensus on Radiotherapy for Glioma similarly recommends a total dose of 54–60 Gy (with fraction sizes of 1.8–2.0 Gy) for large high-grade gliomas or those located in eloquent brain areas (20). A Spanish retrospective study of over 800 high-grade glioma patients receiving postoperative radiotherapy suggested that initiating radiotherapy early (recommended within 4–6 weeks after surgery) can effectively prolong survival (21). Evidence indicates that doses exceeding 60 Gy do not improve survival and are associated with a significant decline in quality of life.

While radiotherapy remains the standard treatment for DMG, combinations with novel chemotherapeutic agents are under investigation to improve outcomes. A major obstacle to drug therapy is the blood-brain barrier, a structure composed of endothelial cells, capillaries, and a basement membrane that restricts the passage of most anticancer agents into the brain (22). Furthermore, tumor growth can compress surrounding vasculature, and both radiotherapy and surgery may induce vascular injury, potentially leading to stenosis or occlusion (23). Such occlusion can impair cerebral blood flow or intratumoral metabolic circulation, exacerbating the clinical condition or hampering treatment efficacy. Temozolomide(TMZ) is currently the recommended chemotherapeutic agent for high-grade gliomas. An Italian multicenter retrospective study suggested that although concurrent temozolomide chemoradiation did not significantly prolong overall survival in patients with H3K27-altered DMG, it may help mitigate the risk or severity of vascular or tumor-associated channel occlusion. This effect could contribute to preserving normal brain physiology and improving patient quality of life (24). The combination of temozolomide and radiotherapy has been shown to improve survival rates in some cases. The patient tolerated the entire radiotherapy course well without significant toxicity or side effects.

Considering the unmethylated O^6^-methylguanine-DNA methyltransferase (MGMT) promoter status of our patient’s tumor, which is associated with limited efficacy of temozolomide, the subsequent treatment strategy was modified (25, 26). After evaluating the patient’s young age and favorable performance status—indicative of a capacity to tolerate more intensive chemotherapy—we selected teniposide (VM-26) as an additional agent, leveraging its ability to penetrate the blood-brain barrier. Teniposide is a lipophilic podophyllotoxin derivative whose ability to cross the BBB forms a key pharmacological rationale for its use in brain tumors. As a topoisomerase II inhibitor, it acts by forming a stable ternary complex with the enzyme and DNA, inducing double-strand breaks. This mechanism specifically arrests the proliferation of tumor cells during the S and G2 phases of the cell cycle and induces apoptosis (27). Research involving cell and animal models has demonstrated that combining teniposide with temozolomide causes more significant DNA damage than either agent alone. This synergy is attributed to teniposide’s ability to enhance the ubiquitination of MGMT, thereby reducing MGMT protein levels and increasing tumor cell sensitivity to temozolomide (28). The antitumor activity of teniposide has also been investigated in other malignancies, A phase III randomized controlled trial conducted by Postmus et al. in patients with brain metastases from small-cell lung cancer (SCLC) demonstrated that teniposide monotherapy achieved an intracranial objective response rate of 22%, which was significantly increased to 57% when combined with whole-brain radiotherapy (WBRT) (P<0.001). This study confirmed the favorable blood-brain barrier penetration of teniposide and its definite antitumor activity against intracranial metastatic lesions (29).

Therapies targeting vascular endothelial growth factor (VEGF) inhibit new blood vessel growth, promote the regression of excessive, abnormal tumor vasculature, and thereby suppress tumor progression. Bevacizumab is a monoclonal antibody targeting VEGF, it inhibits tumor angiogenesis, disrupting the tumor’s nutrient supply to curb its growth, as early as 2007, bevacizumab was investigated in clinical trials for recurrent glioblastoma (GBM), demonstrating promising antitumor activity (30). Subsequent studies have established the safety and efficacy of bevacizumab, both as monotherapy and in combination with radiotherapy and chemotherapy. A 2020 study demonstrated that a combination regimen of bevacizumab with chemotherapeutic agents such as etoposide and radiotherapy significantly prolonged progression-free survival in patients with high-grade gliomas (31). Through its mechanisms of anti-angiogenesis and vascular normalization, bevacizumab not only rapidly reduces peritumoral edema and alleviates clinical symptoms but may also enhance perfusion within the tumor microenvironment. This improvement potentially creates more favorable conditions for the delivery of concomitant chemotherapeutic drugs (32). Teniposide, a lipophilic topoisomerase II inhibitor with good central nervous system penetration, effectively targets and eliminates rapidly proliferating tumor cells (27).

This case report demonstrates that an intensive adjuvant regimen centered on teniposide, in combination with temozolomide and bevacizumab, holds promise for the treatment of H3K27M-mutant diffuse midline glioma (DMG). The regimen achieved not only an objective radiological response but also clear clinical benefit, with good patient tolerance. Given the patient’s substantial tumor burden, extensive infiltration, and severe peritumoral edema accompanied by significant neurological symptoms, bevacizumab was added to teniposide-based chemotherapy as an individualized strategy to achieve rapid symptomatic relief and improve quality of life while maintaining tumor control. The management of H3K27M-mutant DMG remains a major challenge in neuro-oncology. This case provides preliminary clinical evidence and valuable insights for future research. However, findings from a single case are inherently limited. As more similar cases are accumulated, we plan to conduct small-sample prospective or retrospective analyses to further evaluate the efficacy and safety of this combination regimen in patients with H3K27M-mutant DMG. Such studies would provide higher-level evidence to support its clinical application and contribute to the development of more effective treatment strategies for this patient population with a notoriously poor prognosis.

## Data Availability

The datasets presented in this study can be found in online repositories. The names of the repository/repositories and accession number(s) can be found in the article/supplementary material.
