# Correction: Promising efficacy of teniposide in H3K27M mutant diffuse midline gliomas: a case report

**DOI:** 10.3389/fonc.2026.1868322

**Published:** 2026-05-11

**Authors:** Xiaoman Wang, Tingting Niu, Junxian Xia, Jing Liu, Mengqi Sun

**Affiliations:** 1 The Second Clinical Medical College of Jinan University, Department of Radiotherapy, Shenzhen People’s Hospital, Shenzhen, Guangdong, China; 2 The Second Clinical Medical College of Jinan University, Department of Pharmacy, Shenzhen People’s Hospital, Shenzhen, Guangdong, China; 3 Departments of Pathology, Shenzhen Second People’s Hospital, The First Affiliated Hospital of Shenzhen University Health Science Center, Shenzhen, Guangdong, China

**Keywords:** chemotherapy, diffuse midline glioma, H3K27M mutation, targeted therapy, teniposide

An incorrect **Funding** statement was provided. The correct funder is “Sanming Project of Medicine in Shenzhen(Nos. SZSM202411003) and the Science, Technology & Innovation Commission of Shenzhen Municipality (Nos. JCYJ20240813104127037).”

The original version of this article has been updated.

